# Selective Detection of NO_2_ Using Cr-Doped CuO Nanorods

**DOI:** 10.3390/s120608013

**Published:** 2012-06-11

**Authors:** Kang-Min Kim, Hyun-Mook Jeong, Hae-Ryong Kim, Kwon-Il Choi, Hyo-Joong Kim, Jong-Heun Lee

**Affiliations:** Department of Materials Science and Engineering, Korea University, Seoul 136-713, Korea; E-Mails: mackjan@korea.ac.kr (K.-M.K.); hyonez@naver.com (H.-M.J.); kimryong@gmail.com (H.-R.K.); saaryun@korea.ac.kr (K.-I.C.); yoarin@korea.ac.kr (H.-J.K.)

**Keywords:** CuO, Cr_2_O_3_, p-type, gas sensor, nanostructures, nanosheets, nanorods

## Abstract

CuO nanosheets, Cr-doped CuO nanosheets, and Cr-doped CuO nanorods were prepared by heating a slurry containing Cu-hydroxide/Cr-hydroxide. Their responses to 100 ppm NO_2_, C_2_H_5_OH, NH_3_, trimethylamine, C_3_H_8_, and CO were measured. For 2.2 at% Cr-doped CuO nanorods, the response (*R_a_/R_g_*, *R_a_*: resistance in air, *R_g_*: resistance in gas) to 100 ppm NO_2_ was 134.2 at 250 °C, which was significantly higher than that of pure CuO nano-sheets (*R_a_/R_g_* = 7.5) and 0.76 at% Cr-doped CuO nanosheets (*R_a_/R_g_* = 19.9). In addition, the sensitivity for NO_2_ was also markedly enhanced by Cr doping. Highly sensitive and selective detection of NO_2_ in 2.2 at% Cr-doped CuO nanorods is explained in relation to Cr-doping induced changes in donor density, morphology, and catalytic effects.

## 1. Introduction

Oxide semiconductors have been used to detect oxidizing and reducing gases in a simple and cost-effective manner [1–3]. Their chemiresistive variation emanates from the oxidative or reductive interaction of the analyte gas with the oxide semiconductor surface and the consequent change in the charge carrier concentration. In n-type oxide semiconductor gas sensors such as those comprising SnO_2_, ZnO, TiO_2_, WO_3_, and In_2_O_3_, the electron depletion layer is formed by the adsorption of negatively charged oxygen, which dominates the overall conduction process [4]. In contrast, for p-type oxide semiconductor gas sensors such as those comprising CuO, NiO, Co_3_O_4_, and Cr_2_O_3_, the adsorption of negatively charged oxygen forms a hole accumulation layer near the surface. Thus, conduction occurs along the conductive hole accumulation layer [5].

Various strategies aimed at enhancing gas sensing characteristics such as gas response and selectivity have been reported for n-type oxide semiconductors, which include control of the grain size [6,7], morphologies [8], charge carrier concentration [9], catalytic additive(s) [10], and inter-nanostructure contacts [11] of the sensing materials. The results of these studies suggest that selecting additives that can change the charge carrier concentration, catalytic function, and morphology is very advantageous for enhancing or modifying their gas sensing characteristics. However, studies on the gas sensing characteristics of p-type oxide semiconductors are in their early stages, and most researchers have reported on the gas sensing characteristics of pure p-type oxide semiconductors without additives. For example, the design of highly sensitive and selective CuO sensors using oxide additives has barely been investigated. In contrast, the gas sensing characteristics of various undoped CuO nanostructures including thin films [12,13], nanoparticles [14–16], nanowires [17–19], nanorods [20], nanoribbons [21], nanosheets [15], worm-like structures [22], and hierarchical structures [23] have been studied extensively.

In this study, highly crystalline CuO nanosheets, Cr-doped CuO nanosheets, and Cr-doped CuO nanorods were prepared by a facile chemical route without using a surfactant or capping agent, and their gas sensing characteristics were studied. The doping of CuO nanostructures with Cr significantly enhanced their response and selectivity toward NO_2_. The main focus of this study was investigating the reasons for the enhanced response and selectivity toward NO_2_ in relation to the Cr-doping-induced changes in the morphology, surface area, resistance in air, and catalytic property.

## 2. Experimental Section

### 2.1. Preparation of CuO and Cr-Doped CuO Nanostructures

CuO nanosheets were prepared by the following procedure: CuCl_2_·2H_2_O (17.05 g, >99%, Kanto Chemical, Japan) was dissolved in deionized water (100 mL). Then, 50% NaOH aqueous solution (32 g, Samchun Chemical, Korea) was instantaneously poured into the solution, which caused the precipitation of bright blue Cu hydroxide. The precipitate slurry was heated to 100 °C at a heating rate of 1.67 °C/min. Then, the temperature of the slurry solution was maintained at 100 °C for 30 min. During the reaction, the bright blue Cu-hydroxide precipitate was converted into dark brown CuO nanosheets. CuO nanostructures doped with Cr were prepared by the following procedure: CuCl_2_·2H_2_O (17.05 g) and CrCl_3_·6H_2_O (0.267 g or 0.799 g, >98%, Aldrich, USA) were dissolved in deionized water (100 mL). 50% NaOH aqueous solution (32 g) was instantaneously poured into the solution. The hydroxide precursors were converted into dark brown Cr-doped CuO nanostructures by heating the precipitate slurry solution at 100 °C for 30 min. Cr-doped CuO nanosheets and nanorods were prepared from solutions containing low and high concentrations of CrCl_3_·6H_2_O ([Cr^3+^]/([Cu^2+^]+ [Cr^3+^]) = 0.99 at% and 2.9 at%), respectively. The Cr concentrations of the Cr-doped CuO nanosheets and nanorods were determined by inductively coupled plasma mass spectroscopy to be 0.76 at% and 2.2 at%, respectively. For simplicity, herein the two Cr-doped nanostructures will be referred to as 0.76Cr-CuO and 2.2Cr-CuO specimens.

### 2.2. Characterization

The phase and crystallinity of the powders were analyzed by X-ray diffraction (XRD, Rigaku D/MAX-2500 V/PC, Rigaku, Japan). The morphology of the powders was investigated by field-emission scanning electron microscopy (FE-SEM, S-4800, Hitachi Co. Ltd., Japan). High resolution transmission electron microscopy (HR-TEM, JEM-2100F, JEOL Co. Ltd., Japan) was used to examine the microstructure of the CuO nanostructures. The surface areas were measured by the Brunauer-Emmett-Teller method (Tristar 3000, Micromeritics Co. Ltd., USA).

### 2.3. Gas Sensing Characteristics

The as-prepared CuO and Cr-doped nanostructures were heated at 500 °C for 1 *h* in order to dehydrate the residual hydroxide and to increase the thermal stability of each sensing material at the sensor temperature (250–400 °C). The CuO and Cr-doped nanostructures were dispersed in distilled water (nanosturctures: water = 1:9 by weight) and the slurry was applied to an alumina substrate (size: 1.5 mm × 1.5 mm, thickness: 0.25 mm) using micro-pipette. The alumina substrate was comprised of two Au electrodes (electrode width: 1 mm, electrode spacing: 0.2 mm). After drying, the sensor element was heated-treated again at 500 °C for 1 *h* to remove the solvent. The sensor was then placed in a quartz tube and the temperature of the furnace was stabilized at 400 °C. A flow-through technique with a constant flow rate of 500 cm^3^/min was used and a 4-way valve was employed to switch the gas atmospheres. The gas responses (S = *R_a_/R_g_* for oxidizing gas or *R_g_/R_a_* for reducing gas, *R_a_*: resistance in dry air, *R_g_*: resistance in gas) to 100 ppm NO_2_, C_2_H_5_OH, NH_3_, trimethylamine(TMA), C_3_H_8_, and CO were measured over the range 250–400 °C. Gases at 100 ppm NO_2_, C_2_H_5_OH, NH_3_, TMA, C_3_H_8_, and CO all on a dry air balance were used as parent gases for the measurements. The reproducibilities in sensor resistance and gas response were confirmed by measuring the sensing characteristics of 2–3 sensors for each sensor condition. The concentration of NO_2_ was controlled from 5–100 ppm by changing the mixing ratio of the parent gases (100 ppm NO_2_, dry air balance) and dry synthetic air. The dc 2-probe resistance of each sensor was measured using an electrometer that was interfaced with a computer.

## 3. Results and Discussion

**Figure 1.**
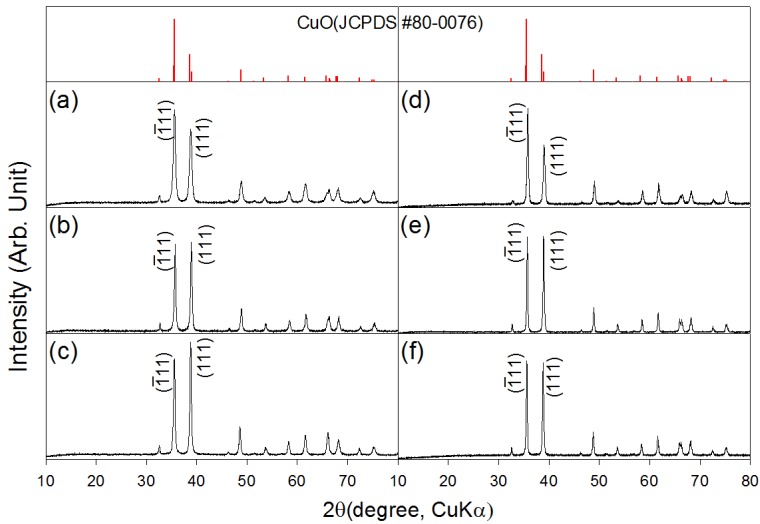
XRD patterns of CuO and Cr-doped CuO nanostructures. (**a**) as-prepared CuO nanosheets; (**b**) as-prepared 0.76Cr-CuO nanosheets; (**c**) as-prepared 2.2Cr-CuO nanorods, (**d**) CuO nanosheets after heat treatment at 500 °C for 1 *h*, (**e**) 0.76Cr-CuO nanosheets after heat treatment at 500 °C for 1 *h*, and (**f**) 2.2Cr-CuO nanorods after heat treatment at 500 °C for 1 *h*.

XRD patterns of the as-prepared and heat treated CuO nanostructures are shown in Figure 1. The CuO nanosheets, 0.76Cr-CuO nanosheets, and 2.2Cr-CuO nanorods showed monoclinic CuO phases (JCPDS #80-0076), indicating that the undoped or Cr-doped Cu-hydroxide slurry solutions were completely dehydrated into CuO by heating at 100 °C (Figure 1(a–c)). Regardless of the sample type, the CuO phase remained unchanged after heat treatment at 500 °C (Figure 1(d–f)). No Cr_2_O_3_ phase was found both in the 0.76Cr-CuO nanosheets, and 2.2Cr-CuO nanorods. Note that the relative intensities of the (ī11) and (111) peaks depended on the degree of Cr doping. After heat treatment at 500 °C for 1 *h*, the intensity ratios between the (111) peak at 2θ = 38.8° and the (ī11) peak at 2θ = 35.5° were 0.64, 0.98, and 0.99 for the CuO nanosheets, 0.76Cr-CuO nanosheets, and 2.2Cr-CuO nanorods, respectively. The morphological change from CuO nanosheets to 2.2Cr-CuO nanorods seems to relate to the Cr-doping-induced change of preferred orientation although its origin remains unclear.

**Figure 2.**
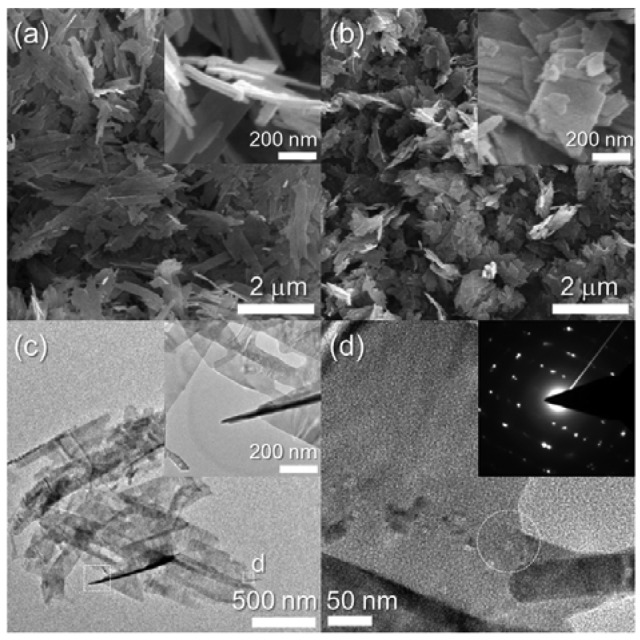
SEM and TEM images of CuO nanosheets. (**a**) SEM image of as-prepared CuO nanosheets, (**b**) SEM image of CuO nanosheets after heat treatment at 500 °C for 1 *h*, and (**c,d**) TEM images and SAED pattern of CuO nanosheets after heat treatment at 500 °C for 1 *h*.

Dehydration of the Cu-hydroxide precipitate solution led to the formation of CuO nanosheets. The nanosheets were 2–3 μm long, 150–250 nm wide, and ∼20 nm thick (inset, Figure 2(a)). The morphology and size of the nanosheets remained similar after heat treatment at 500 °C (Figure 2(b)). The sheet-like morphology was confirmed again using TEM analysis (Figure 2(c)). Selected area electron diffraction in the circled region in Figure 2(d) clearly indicates the presence of a highly crystalline CuO structure.

**Figure 3.**
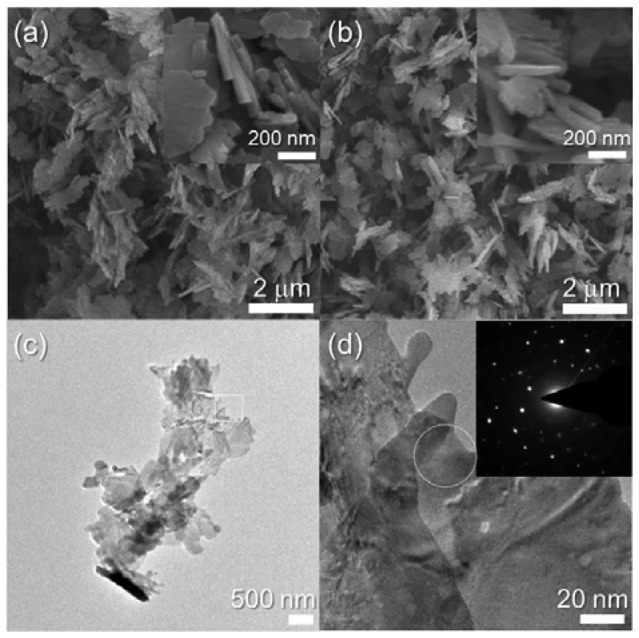
SEM and TEM images of 0.76Cr-CuO nanosheets. (**a**) SEM image of as-prepared 0.76Cr-CuO nanosheets, (**b**) SEM image of 0.76Cr-CuO nanosheets after heat treatment at 500 °C for 1 *h*, and (**c,d**) TEM images and SAED pattern of 0.76Cr-CuO nanosheets after heat treatment at 500 °C for 1 *h*.

Nanosheets were also obtained in the 0.76 at% Cr-doped CuO specimen (Figure 3(a)). The edge width of the as-prepared 0.76Cr-CuO nanosheets was similar to that of the CuO nanosheets (*cf*. Figures 2(a) and 3(a)). In comparison, the morphologies of the nanosheets became more irregular and slightly thicker upon Cr doping (insets in Figures 2(a) and 3(a)). The morphology of the 0.76Cr-CuO nanosheets remained similar after heat treatment at 500 °C (Figure 3(b)) and the nanosheets were identified as being crystalline by TEM analyses (Figure 3(c,d)).

**Figure 4.**
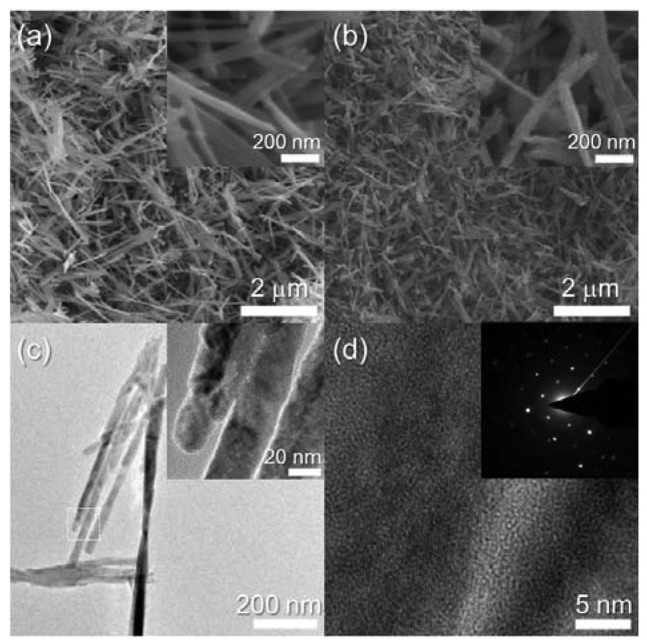
SEM and TEM images of 2.2Cr-CuO nanorods. (**a**) SEM image of as-prepared 2.2Cr-CuO nanorods, (**b**) SEM image of 2.2Cr-CuO nanorods after heat treatment at 500 °C for 1 *h*, and (**c,d**) TEM images and SAED pattern of 2.2Cr-CuO nanorods after heat treatment at 500 °C for 1 *h*.

Different morphologies of the CuO nanostructures occurred in the 2.2Cr-CuO specimen (Figure 4(a)). The 2.2Cr-CuO nanorods were 2–3 μm long, 30 nm thick and the morphology did not change after heat treatment at 500 °C (Figure 4(b)). The 2.2Cr-CuO nanorods after heat treatment were also identified as being highly crystalline ones by their TEM images and selected area electron diffraction patterns (Figure 4(c,d)).

**Figure 5.**
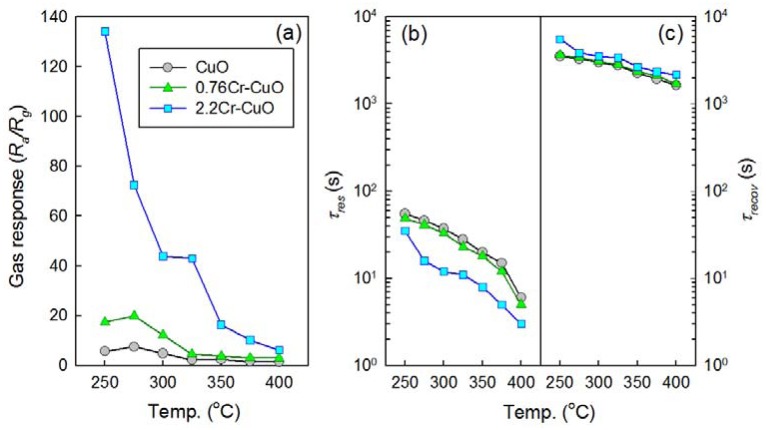
(**a**) Gas responses (*R_a_/R_g_*), (**b**) 90% response time (*τ_res_*), and (**c**) 90% recovery time (*τ_recov_*) of CuO, 0.76Cr-CuO, and 2.2Cr-CuO sensors to 100 ppm NO_2_ over the range 250–400 °C.

The gas sensing characteristics of the CuO nanosheets, 0.76Cr-CuO nanosheets, and 2.2Cr-CuO nanorods showed decreases in resistance upon exposure to oxidizing gases such as NO_2_ and increases in resistance upon exposure to reducing gases such as C_2_H_5_OH, NH_3_, TMA, C_3_H_8_, and CO. This is consistent with the gas sensing characteristics of p-type oxide semiconductors. Thus, the *R_a_/R_g_* (*R_a_*: resistance in air; *R_g_*: resistance in gas) and *R_g_/R_a_* were defined as the gas responses to oxidizing and reducing gases, respectively. Below the sensor temperature of 250 °C, it was difficult to measure gas sensing characteristics due to the sluggish recovery kinetics. Thus, the gas responses (*R_a_/R_g_*) to 100 ppm NO_2_ were measured at sensor temperatures over the range 250–400 °C (Figure 5(a)). The responses of the CuO nanosheets to 100 ppm NO_2_ exhibited a maximum value (*R_a_/R_g_* = 7.5) at 275 °C and decreased to 1.4 as the sensor temperature increased. The doping of 0.76 at% Cr enhanced the maximum NO_2_ responses to 19.9 at 275 °C. This corresponded to a 2.65-fold increase in sensitivity. The NO_2_ responses of the 2.2Cr-CuO nanorods were significantly higher than those of CuO and the 0.76Cr-CuO nanosheets over the entire sensor temperature range. The NO_2_ response of the 2.2Cr-CuO nanorods was as high as 134.2 at 250 °C and decreased monotonically to 6.2 as the sensor temperature increased up to 400 °C. The times to reach 90% variation of resistance upon exposure to gas and air, the 90% response and recovery times (*τ_res_* and *τ_recov_*), were determined from the sensing transients (Figure 5(b,c)). The *τ_res_* values of CuO sensors decreased from 55 *s* to 6 *s* as the sensor temperature increases (Figure 5(b)). Note that the *τ_res_* values tend to decrease as increasing Cr doping concentration at the same sensor temperature. This indicates that the reaction kinetics can be enhanced either by thermal activation of sensing reaction or by the catalytic promotion. In contrast, very long recovery times (1,642–5,536 *s*) were required (Figure 5(c)).

**Figure 6.**
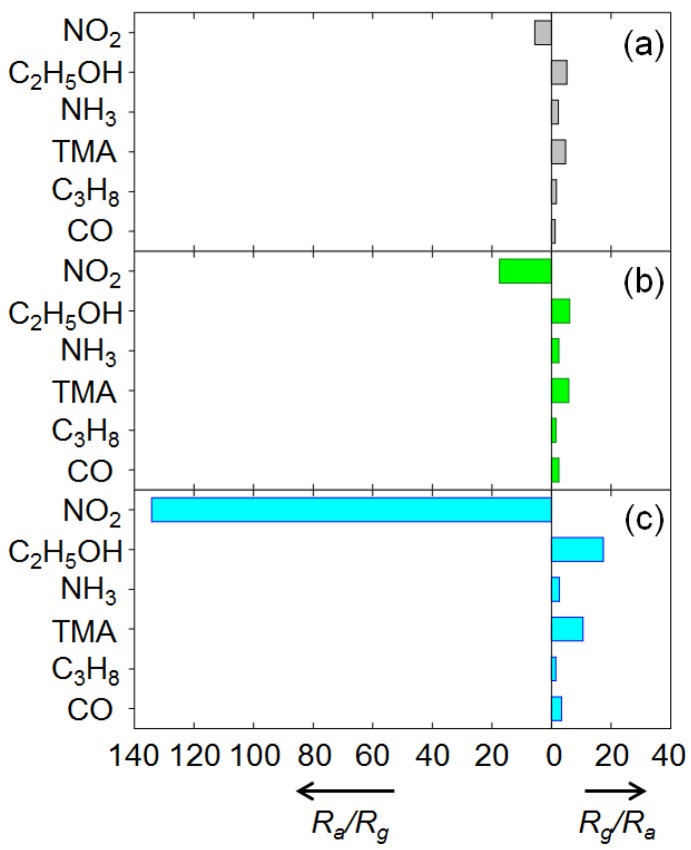
Gas responses (*R_a_/R_g_* or *R_g_/R_a_*) of (**a**) CuO, (**b**) 0.76Cr-CuO and (**c**) 2.2Cr-CuO nanostructures to 100 ppm NO_2_, C_2_H_5_OH, NH_3_, TMA, C_3_H_8_, and CO at 250 °C.

The responses to 100 ppm NO_2_, C_2_H_5_OH, NH_3_, TMA, C_3_H_8_, and CO at 250 °C are shown in Figure 6. For the CuO nanosheets, the response (*R_a_/R_g_*) to 100 ppm NO_2_ was 5.6, while the responses (*R_g_/R_a_*) to other gases ranged from 1.2–5.1 (Figure 6(a)). In particular, the responses to 100 ppm C_2_H_5_OH (*R_g_/R_a_* = 5.1) and TMA (*R_g_/R_a_* = 4.7) were comparable to that for 100 ppm NO_2_ although the resistance of the sensors varied in the opposite direction. By doping with 0.76 at% Cr, the response (*R_a_/R_g_*) to 100 ppm NO_2_ increased to 17.5, while the responses to the other gases remained similar (*R_g_/R_a_* = 1.5–6.1) (Figure 6(b)). This suggests that NO_2_ can be detected in a more selective manner using 0.76Cr-CuO nanosheets.

The response (*R_a_/R_g_*) to 100 ppm NO_2_ was markedly enhanced to 134.2. The responses to other gases except C_2_H_5_OH, TMA, and CO remained similar (*R_g_/R_a_* = 1.5–2.7). Although the responses to 100 ppm C_2_H_5_OH, TMA, and CO were also increased, the 3.4-fold enhancement of the C_2_H_5_OH response (17.4/5.1 = 3.4), the 2.3-fold enhancement of the TMA response (10.6/4.7 = 2.3), and the 2.8-fold enhancement of the CO response (3.4/1.2 = 2.8) were negligibly small compared to the 24.1-fold enhancement of the NO_2_ response (134.2/5.6 = 24.1). This clearly verifies that 2.2 at% Cr doping of the CuO nanostructures resulted in highly selective and sensitive detection of NO_2_ in the presence of interfering gases.

**Figure 7.**
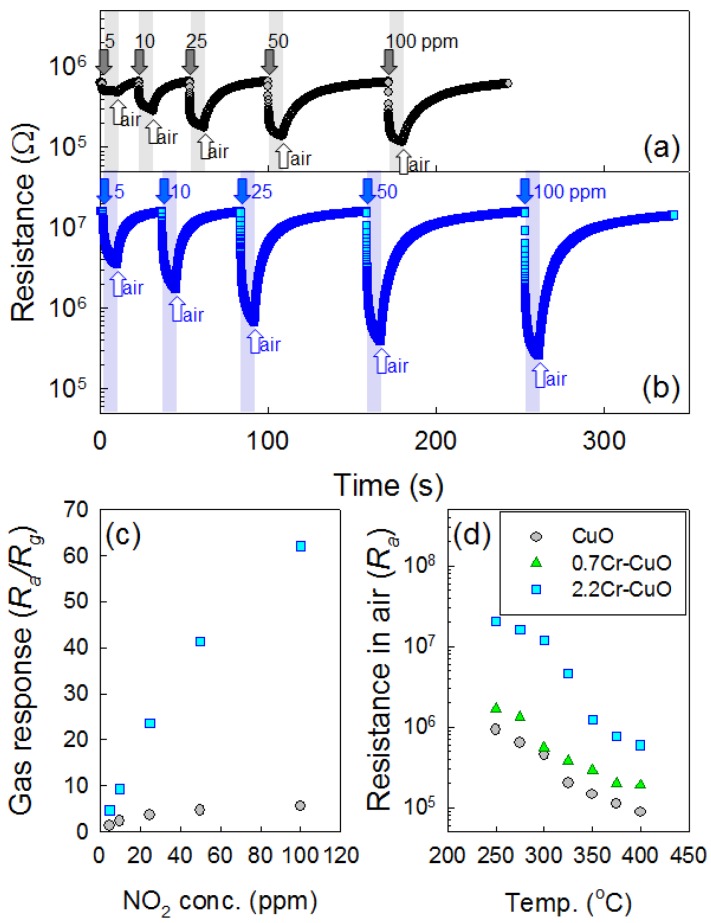
(**a**) Sensing transients of CuO nanostructures over the range 5–100 ppm NO_2_ at 275 °C, (**b**) Sensing transients of 2.2Cr-CuO nanostructures over the range 5–100 ppm NO_2_ at 275 °C, (**c**) Gas responses (*R_a_/R_g_*) of CuO and 2.2Cr-CuO nanostructures over the range 5–100 ppm NO_2_ at 275 °C, and (**d**) resistances in air (*R_a_*) of the CuO, 0.76Cr-CuO, and 2.2Cr-CuO nanostructures over the range 250–400 °C.

Although the 2.2Cr-CuO nanorods showed the highest response to NO_2_ at 250 °C, the responses and recoveries of the sensors were sluggish. For example, the 90% response time upon exposure to 100 ppm NO_2_ at 250 °C was ∼10 min. Accordingly, the operation temperature of the sensor was elevated slightly to 275 °C to enhance the sensing speed without significantly sacrificing the NO_2_ response. The dynamic sensing transients of the CuO nanosheets and 2.2Cr-CuO nanorods over the range 5–100 ppm NO_2_ at 275 °C (Figure 7(a,b)) showed decreases in their resistances and recoveries upon exposure to NO_2_ and air, respectively. The responses over the range 5–100 ppm NO_2_ of the 2.2Cr-CuO nanorods were in the range 4.6–62.1, while those of the undoped CuO nanosheets were in the range of 1.3–5.5. The NO_2_ response values of the 2.2Cr-CuO nanorods in the present study were among the highest values reported in the literature for CuO nanostructures [15,18,19]. This confirms again that Cr doping is very effective for enhancing the NO_2_ response of CuO (Figure 7(c)).

The gas response and selectivity of oxide semiconductors are related to various parameters such as their dimensions [6,7], morphologies of the oxide nanostructures [8], donor concentrations [9], charge carrier depletion/accumulation near the surface [4,5], sensing material composition [24,25], and catalyst loading [10]. In the present study, the variations in morphology, charge carrier concentrations, and catalytic effects due to Cr doping were found to change the gas sensing characteristics of the CuO nanostructures.

**Figure 8.**
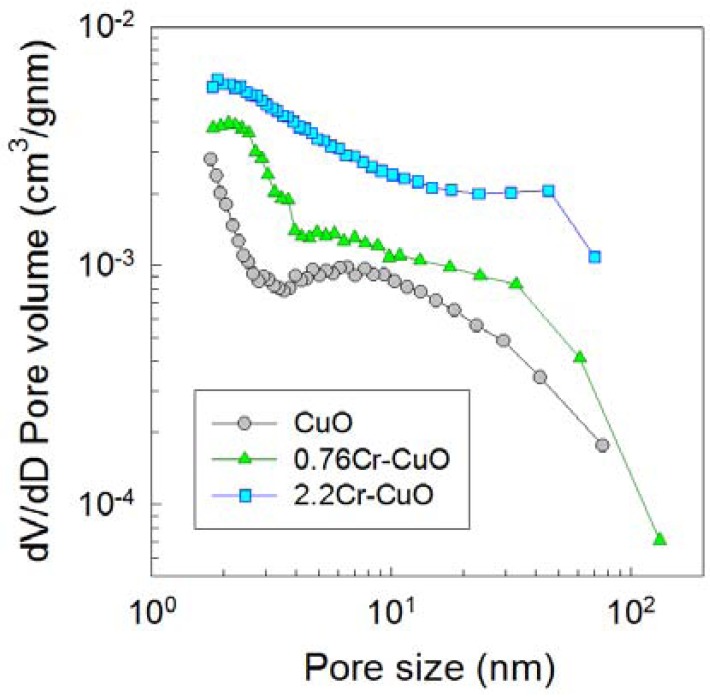
Pore size distributions of CuO, 0.76Cr-CuO, and 2.2Cr-CuO nanostructures determined from nitrogen adsorption-desorption isotherm.

The pore sizes, volume distributions, and surface areas were analyzed by measuring nitrogen adsorption-desorption isotherms (Figure 8). The surface areas of the CuO, 0.76Cr-CuO, and 2.2Cr-CuO nanostructures after heat treatment at 500 °C for 1 *h* were 19.8, 22.3, and 42.3 m^2^/g, respectively. Note that the pore volumes over the entire pore size range increased with an increase in the Cr doping concentration, indicating that the increase in both the surface area to volume ratio and gas accessibility due to Cr doping can be considered as one of the important reasons to enhance the gas response of the CuO nanostructures.

In order to investigate the incorporation of Cr into the CuO nanostructures and its consequent effect on the charge carrier concentrations, the resistances in air (*R_a_*) of the sensors were measured as a function of sensor temperature (Figure 7(d)). The *R_a_* values of all three sensors showed monotonic decreases with increasing temperature, indicating that the thermally activated generation of holes occurred. At the constant sensing temperature, the *R_a_* values increased with increased doping concentration of Cr (Figure 7(d)) and these tendencies were reproducible even when considering the fluctuation in the resistances of the sensors. The ionic radius of Cu^2+^ at the coordination numbers (CN) of 4 and 6 are 0.71 Å and 0.87 Å, respectively [26]. Although the ionic radius Cr^3+^ at CN = 4 in not available in the literature, it is likely to be smaller than Cu^2+^ at CN = 4 considering the ionic radius of Cr^3+^ at CN = 6 (0.755 Å) [26]. High *R_a_* values and the absence of Cr_2_O_3_ peak in the 2.2Cr-CuO nanorods strongly suggests the incorporation of Cr into the CuO lattice, although further study is necessary to confirm this. However, the possibility for the presence of small concentration of Cr_2_O_3_ or Cr-containing second phase below the detection limit of X-ray diffraction cannot be excluded. In p-type oxide semiconductors, the adsorption of negatively charged oxygen results in the formation of a hole accumulation layer near the surface [5]. The reaction between reducing gases and negatively charged surface oxygen releases the electrons, which thins the hole accumulation layer via electron-hole recombination and increases the resistance of the sensor. In contrast, the resistance of the sensor becomes lower upon exposure to oxidizing gas due to the thickening of the hole accumulation layer. If the hole accumulation layer in air is relatively thick, the variation in resistance of the sensor upon exposure to oxidizing gas or reducing gas should not be high because the thicker or less thick charge accumulation layers near the surface do not cause significant changes in conduction. In contrast, if the charge accumulation layer in air is very thin, thickening or further thinning of the hole accumulation layer upon exposure to oxidizing and reducing gases will lead to relatively high variation in the–resistance of the sensor. From this viewpoint, the enhancement of responses to NO_2_ and reducing gases by Cr doping as shown in Figure 5 can be explained in part by the incorporation of Cr^3+^ into the CuO lattice and the consequent decrease in its charge carrier (hole) concentration.

The effect of Cr doping on the selectivity of NO_2_ detection was also assessed. From the results shown in Figure 5, the ratio between the response to 100 ppm NO_2_ and that to other gases (*S_NO2_/S_gas_* = (*R_a_/R_NO2_*)/(*R_gas_/R_a_*)) was calculated as a measure of NO_2_ selectivity. The *S_NO2_/S_gas_* values (gases: 100 ppm C_2_H_5_OH, NH_3_, TMA, C_3_H_8_, and CO) of the 2.2Cr-CuO nanorods ranged from 7.7 to 91.3, which were significantly higher than those of the pure CuO nanosheets (1.1–4.7) and the pure CuO nanosheets (2.9–7.0). This strongly indicates that Cr oxide acts as a catalyst to promote the NO_2_ sensing reaction. NO_2_ is known to adsorb onto the surface of Cr_2_O_3_, and it dissociates into NO and oxygen [27]. Considering that the adsorption of NO_2_ in the form of NO^−^ or NO_2_^−^ leads to chemiresistive variation in oxide semiconductors [28–30], the promotion of NO_2_ sensing reaction can be explained in part by the enhanced NO_2_ dissociation reaction due to the catalytic function of the Cr-component or Cr-containing second phase below the detection limit of X-ray diffraction. When a single component gas is measured using semiconductor gas sensors, the oxidizing gas such as NO_2_ and other reducing gases can be easily discriminated due to their opposite direction of chemiresistive variation. However, when the mixture between oxidizing and reducing gases should be measured, the situation becomes more complex. As a representative example for this issue will be ‘air quality sensor’ to control air intake from outside to the automotive cabin in an automatic manner. In general, CO and NO_2_ are measured to determine the presence of pollutant emitting gasoline and diesel vehicles, respectively [31]. In p-type oxide semiconductors, when both of diesel and gasoline engines emit NO_2_ and CO, the decrease of sensor resistance by NO_2_ can be nullified by the increase of sensor resistance by CO, which can lead to the malfunction of the sensor. Accordingly, the 24.1-fold enhancement of the NO_2_ response by Cr-doping in the present study, which is significantly higher than the 2.8-fold enhancement of the CO, can be used to detect the presence of pollutant emitting diesel vehicles with the minimum disturbance by CO emitted from gasoline vehicles. The increase in the *S_NO2_/S_gas_* values due to Cr doping in the present study, accordingly, can enable the selective detection of NO_2_ with minimum cross-responses from reducing gases.

Finally, the long-term stability of sensor is very important for the real applications. Sysoev *et al.* [32] reported that SnO_2_ nanowires prepared at 950 °C by vapor-solid method are more stable during long-term sensor operation at 300 °C than SnO_2_ nanoparticles (diameter: ∼4 nm) heat treated at 400 °C. Considering the small size of SnO_2_ nanoparticles and low heat treatment temperature, the degradation of sensing characteristics in SnO_2_ nanoparticles can be attributed to the sintering and aggregation between primary particles. In the present study, the morphologies of nanosheets or nanorods in Cr-doped CuO nanorods remained similar even after heat treatment at 500 °C. Moreover, taking into account that the optimum sensor temperature for NO_2_ detection (250–275 °C) is significantly lower than the temperature for heat treatment (500 °C), the present sensor can be regarded as thermally stable although the degradation of sensor by long-term exposure to NO_2_ should be examined further.

## 4. Conclusions

Various morphologies of highly crystalline pure and Cr-doped CuO nanostructures such as CuO nanosheets, Cr-doped CuO nanosheets, and Cr-doped CuO nanorods were prepared by heating slurry solutions containing Cu-hydroxide/Cr-hydroxide, and their gas sensing characteristics were investigated. Doping CuO nanostructures with 2.2 at% Cr enhanced the NO_2_ response to 24.1 times and significantly increased the NO_2_ selectivity. Enhancement of the NO_2_ response and selectivity of 2.2 at% Cr-doped CuO nanorods was attributed to increase in surface area and pore volume, the electronic sensitization due to the incorporation of Cr into the CuO lattice, and the catalytic function of Cr. Cr-doped CuO nanorods are an effective solution to detect NO_2_ in a selective manner with minimum cross-responses to C_2_H_5_OH, NH_3_, trimethylamine, C_3_H_8_ and CO.

## References

[1] Yamazoe N. (2005). Toward innovations of gas sensor technology. Sens. Actuators B Chem..

[2] Franke M.E., Koplin T.J., Simon U. (2006). Metal and metal oxide nanoparticles in chemiresistors: Does the nanoscale matter?. Small.

[3] Shimizu Y., Egashira M. (1999). Basic aspects and challenges of semiconductor gas sensors. MRS Bull..

[4] Barsan N., Weimar N. (2001). Conduction model of metal oxide gas sensors. J. Electroceram..

[5] Pokhrel S., Simon C.E., Quemener V., Barsan N., Weimar U. (2008). Investigation of conduction mechanism in Cr_2_O_3_ gas sensing thick films by ac impedance spectroscopy and work function changes measurement. Sens. Actuators B Chem..

[6] Xu C.N., Tamaki J., Miura N., Yamazoe N. (1991). Grain size effects on gas sensitivity of porous SnO_2_-based elements. Sens. Actuators B Chem..

[7] Rothschild A. (2004). The effect of grain size on the sensitivity of nanocrystalline metal-oxide gas sensors. J. Appl. Phys..

[8] Lee J.-H. (2009). Gas sensors using hierarchical and hollow oxide nanostructures: Overview. Sens. Actuators B Chem..

[9] Yamazoe N., Shimanoe K. (2009). New perspective of gas sensor technology. Sens. Actuators B Chem..

[10] Kim S.-J., Hwang I.-S., Na C.W., Kim I.-D., Kang Y.C., Lee J.-H. (2011). Ultrasensitive and selective C_2_H_5_OH sensors using Rh-loaded In_2_O_3_ hollow spheres. J. Mater. Chem..

[11] Hwang I.-S., Kim Y.-S., Kim S.-J., Ju B.-K., Lee J.-H. (2009). A facile fabrication of semiconductor nanowires gas sensor using PDMS patterning and solution deposition. Sens. Actuators B Chem..

[12] Singh I., Bedi R.K. (2011). Studies and correlation among the structural, electrical and gas response properties of aerosol spray deposited self-assembled nanocrystalline CuO. Appl. Surf. Sci..

[13] Barreca D., Comini E., Gasparotto A., Maccato C., Sada C., Sberveglieri G., Tondello E. (2009). Chemical vapor deposition of copper oxide films and entangled quasi-1D nanoarchitectures as innovative gas sensors. Sens. Actuators B Chem..

[14] Singh I., Bedi R.K. (2011). Surfactant-assisted synthesis, characterizations, and room temperature ammonia sensing mechanism of nanocrystalline CuO. Solid State Sci..

[15] Li Y., Liang J., Tao Z., Chen J. (2008). CuO particles and plates: Synthesis and gas-sensor application. Mater. Res. Bull..

[16] Aslani A., Oroojpour V. (2011). CO gas sensing of CuO nanostructures, synthesized by an assisted solvothermal wet chemical route. Physica B.

[17] Li X., Wang Y., Lei Y., Gu Z. (2012). Highly sensitive H_2_S sensor based on template-synthesized CuO nanowires. RSC Adv..

[18] Li D., Hu J., Wu R., Lu J.G. (2010). Conductometric chemical sensor based on individual CuO nanowires. Nanotechnology.

[19] Kim Y.-S., Hwang I.-S., Kim S.-J., Lee C.-Y., Lee J.-H. (2008). CuO nanowire gas sensors for air quality control in automotive cabin. Sens. Actuators B Chem..

[20] Yang C., Su X., Xiao F., Jian J., Wang J. (2011). Gas sensing properties of CuO nanorods synthesized by a microwave-assisted hydrothermal method. Sens. Actuators B Chem..

[21] Gou X., Wang G., Yang J., Park J., Wexler D. (2008). Chemical synthesis, characterization, and gas sensing performance of copper oxide nanoribbons. J. Mater. Chem..

[22] Liu X., Zhang J., Kang Y., Wu S., Wang S. (2012). Brochantite tabular microspindles and their conversion to wormlike CuO structures for gas sensing. CrystEngComm.

[23] Zhu G., Xu H., Xiao Y., Liu Y., Yuan A., Shen X. (2012). Facile fabrication and enhanced sensing properties of hierarchically porous CuO architectures. ACS Appl. Mater. Eng..

[24] Kim K.-W., Cho P.-S., Kim S.-J., Lee J.-H., Kang C.-Y., Kim J.-S., Yoon S.-J. (2007). The selective detection of C_2_H_5_OH using SnO_2_-ZnO thin film gas sensors prepared by combinatorial solution deposition. Sens. Actuators B Chem..

[25] Kim S.-J., Hwang I.-S., Kang Y.C., Lee J.-H. (2011). Design of selective gas sensors using additive-loaded In_2_O_3_ hollow spheres prepared by combinatorial hydrothermal reactions. Sensors.

[26] Lide D.R. (2005). References. Handbook of Chemistry and Physics.

[27] Xu C., Hassel M., Kuhlenbeck H., Freund H.-J. (1991). Adsorption and reaction on oxide surfaces: NO, NO_2_ on Cr_2_O_3_ (111)/Cr(110). Surf. Sci..

[28] Baratto C., Comini E., Faglia G., Sberveglieri G., Zha M., Zappettini A. (2005). Metal oxide nano-crystals for gas sensing. Sens. Actuators B Chem..

[29] Yamazoe N., Shimanoe K. (2008). Theory of power laws for semiconductor gas sensors. Sens. Actuators B Chem..

[30] Epifani M., Prades J.D., Comini E., Pellicer E., Avella M., Sicilliano P., Faglia G., Cirera A., Scotti R., Morazzoni F., Morante J.R. (2008). The role of surface oxygen vacancies in the NO_2_ sensing properties of SnO_2_ nanocrystals. J. Phys. Chem..

[31] Nakagawa H., Okazaki S., Asakura S., Fukuda F., Akimoto H., Takahashi S., Shigemori A. (2008). An automated car ventilation system. Sens. Actuators B Chem..

[32] Sysoev V.V., Schneider T., Goschnick J., Kiselev I., Habicht W., Hahn H., Strelcov E., Kolmakov A. (2009). Percolating SnO_2_ nanowire network as a stable gas sensor: Direct comparison of long-term performance *versus* SnO_2_ nanoparticle films. Sens. Actuators B Chem..

